# Valproic Acid Reduces Neuroinflammation to Provide Retinal Ganglion Cell Neuroprotection in the Retina Axotomy Model

**DOI:** 10.3389/fcell.2022.903436

**Published:** 2022-05-12

**Authors:** James R. Tribble, Elizabeth Kastanaki, A. Berşan Uslular, Carola Rutigliani, Tim J. Enz, Pete A. Williams

**Affiliations:** Department of Clinical Neuroscience, Division of Eye and Vision, St. Erik Eye Hospital, Karolinska Institutet, Stockholm, Sweden

**Keywords:** neuroinflammation, microglia, astrocyte, retina, glaucoma

## Abstract

Neuroinflammation is a critical and targetable pathogenic component of neurodegenerative diseases, including glaucoma, the leading cause of irreversible blindness. Valproic acid has previously been demonstrated to reduce neuroinflammation and is neuroprotective in a number of experimental settings. To determine whether valproic acid can limit retinal neuroinflammation and protect retinal neurons we used an *ex vivo* retina explant (axotomy) model to isolate resident glial responses from blood-derived monocytes. Neuroinflammatory status was defined using high resolution confocal imaging with 3D morphological reconstruction and cytokine protein arrays. Valproic acid significantly reduced microglia and astrocyte morphological changes, consistent with a reduction in pro-inflammatory phenotypes. Cytokine profiling demonstrated that valproic acid significantly attenuated or prevented expression of pro-inflammatory cytokines in injured retina. This identifies that the retinal explant model as a useful tool to explore resident neuroinflammation in a rapid timescale whilst maintaining a complex system of cell interactions and valproic acid as a useful drug to further explore anti-neuroinflammatory strategies in retinal disease.

## Background

Neuroinflammation is emerging as a critical and targetable pathogenic component of neurodegenerative diseases including Alzheimer’s disease and glaucoma. The anti-epileptic drug valproic acid (VPA) has received renewed interest for its anti-inflammatory properties (Zhang et al., 2008). Across immune cell subtypes, including monocytes, macrophages, and T-cells, VPA has been demonstrated to drive a reduction in proinflammatory cytokine release and reduced expression of immune cell surface receptors (Soria-Castro et al., 2019). In the CNS, VPA has demonstrated both neuroprotective and anti-inflammatory effects in diverse animal models of neurodegenerative conditions including spinal cord injury, traumatic brain injury, stroke, Parkinson’s disease, and optic neuritis (Kim et al., 2007; Liu et al., 2017; Chen S et al., 2018; Chen X et al., 2018; Chang et al., 2019; Kim et al., 2019). Using a comparative transcriptomics approach, we recently identified VPA as a potential modifier of expression of genes common to retinal ganglion cell axon degeneration (Enz, Tribble and Williams, 2021) (the susceptible neuron in many optic neuropathies including glaucoma, Leber’s hereditary optic neuropathy, and autosomal dominant optic atrophy). Pathway analysis of genes revealed neuroinflammatory signaling pathways to be the most commonly shared element and, as hypothesized, VPA provided significant neuroprotection to retinal ganglion cells post-axotomy. Supporting this, VPA has previously been demonstrated to reduce retinal ganglion cell apoptosis in models of glaucoma (Alsarraf et al., 2014; Kimura et al., 2015), a common neurodegenerative disease in which retinal ganglion cells degenerate. A pathogenic feature of glaucoma (in humans and animal models) is progressive retinal ganglion cell degeneration accompanied by progressive neuroinflammation (Williams et al., 2017b). Ourselves and others have identified pro-inflammatory activation of microglia and astrocytes, infiltration of monocytes from the blood, and upregulation of cytokines and neuroinflammatory molecules as key pathological components of glaucoma (Tezel et al., 2010; Bosco, Steele and Vetter, 2011; Breen et al., 2016; Gramlich et al., 2016; Williams et al., 2016; Williams et al., 2019; Cooper et al., 2020; Tribble et al., 2020; Tribble et al., 2021a). Importantly, inhibiting these processes can provide some level of neuroprotection in multiple animal models. However, this neuroprotection is not complete, and often neuroinflammatory strategies provide short-term protection but fail to provide long-term protection. Retina and optic nerve neuroinflammation in glaucoma is highly temporal and context dependent, caveated by the difference in rodent-to-human immune cell sub-types and the ongoing debate of infiltrating monocytes vs proliferative microglia in disease. As such, an anti-neuroinflammatory strategy has yet to make it to glaucoma patients.

Separating resident from infiltrating inflammatory responses in ocular tissue is a non-trivial task. Microglia and tissue macrophages share many common genetic signatures and antigens, making cell specific labeling or sorting difficult without transgenic reporter lines (which are still unlikely to be conclusively accurate and still require verification and refinement in the retina). This approach is not amenable to testing potential anti-inflammatory therapeutics for effects on resident microglia. Thus, we present a modification of the retinal explant model as a simple tool to explore only resident glia activity. Using this model, we demonstrate that VPA significantly reduces microglial morphological changes and significantly attenuates resident glial release of pro-inflammatory cytokines in response to retinal ganglion cell injury.

## Methods

### Animal Strain and Husbandry

All breeding and experimental procedures were performed in accordance with the Association for Research for Vision and Ophthalmology (ARVO) Statement for the Use of Animals in Ophthalmic and Research. Individual study protocols were approved by Stockholm’s Committee for Ethical Animal Research (10389-2018). Male C57BL/6J mice were purchased from The Jackson Laboratory (ME, United States, through SCANBUR AB, Sweden). All mice were housed in a 12 h light/12 h dark cycle with food and drinking water available *ad libitum*. All mice were used at 12-20 weeks of age.

### Retinal Explant Model

The retinal explant axotomy model has been described previously (Tribble et al., 2021c). The model was adapted for the analysis of glial responses (removal of N2 and B27 supplements from the media recipe yielded greater numbers of ramified microglia at 1 day *ex vivo* (*data not shown*)). Mice were euthanized by cervical dislocation, and eyes were enucleated immediately. Retinas were removed and flat mounted onto cell culture inserts ganglion cell layer up (Millicell 0.4 µm pore; Merck). Culture inserts were suspended in 6-well plates so that retinas were supplied by culture media from below. Neurobasal-A media supplemented with 2 mM l-glutamate (GlutaMAX, Gibco) and 1% penicillin/streptomycin (Gibco) was used, and plates were maintained at (37°C, 5% CO_2_) for either 1 or 2 days *ex vivo*. Retinas were treated with either lipopolysaccharide (LPS; from *Salmonella enterica* serotype *typhimurium*, cell culture grade, 100 ng/ml; Sigma) or VPA (valproic acid, sodium salt, 1 mM; Merck Millipore) which was dissolved in the culture media. For controls, eyes were used immediately without culture. All eyes were either fixed in ice-cold PFA (3.7%) in PBS for histology or homogenized for cytokine array at the end time point.

### Immunofluorescent Labelling and Microscopy

Following fixation, retinas were transferred to slides and isolated using a hydrophobic barrier pen. Retinas were permeabilized with 0.5% Triton X-100 (VWR) in PBS for 60 min and blocked in 2% bovine serum albumin (Fisher Scientific) in PBS for 60 min. Primary antibodies were applied overnight at 4°C. The primary antibodies were anti-RBPMS (Rabbit, Novusbio # NBP2-20112, 1:500), anti-Iba1 (Rabbit, Abcam # 178846, 1:500), anti-GFAP (Chicken, Novusbio # NBP1-05198, 1:500), and Isolectin GS-IB_4_ (IsoB4; Invitrogen #I21414, 0.1 mg/ml). Retinas were washed 5 times for 10 min in PBS, and secondary antibodies were applied for 4 h at room temperature. The secondary antibodies were Goat anti-Rabbit AF 568 (Invitrogen # A11011, 1:500), Goat anti-Chicken AF 647 (Abcam # ab150171, 1:500), and Streptavidin AF 488 conjugate (Invitrogen #S11223, 4 μg/ml). Retinas were washed 5 times for 10 min in PBS, DAPI nuclear stain (1 μg/ml in 1M PBS) was applied for 10 min, and tissue washed again once in PBS. Retinas were mounted using Fluoromount-G, glass coverslips were applied, and slides were sealed with nail-varnish.

### Morphological Analysis

RGC density was determined as an average of RBPMS + cells *en face* in six regions of interest per retina, as described previously (Tribble et al., 2021b). Images were acquired on a Leica DMi8 microscope with a CoolLED pE-300 white LED-based light source and a Leica DFC7000 T fluorescence color camera (all Leica). For morphological analysis of microglia and astrocytes, images were acquired on a on a Zeiss LSM800-Airy (20×, image field 532.42 × 532.42 μm, voxel size 0.26 × 0.26 × 0.47 μm). For microglia, four regions of interest were imaged at 1,000 µm from the optic nerve head as *Z*-stacks through the nerve fiber layer to the beginning of the outer nuclear layer to capture all three microglial niches. For astrocytes, *Z*-stacks were acquired through the nerve fiber layer to the beginning of the ganglion cell layer. Microglia images were cropped in *Z* to separate the distinct microglia niches using the nuclear layers as a guide. Microglia morphology was reconstructed semi-automatically using Imaris (Bitplane, version 9.3.2), and individual microglia were analyzed. Number of filaments (processes), total filament (process) length, filament (field) area, and filament volume were calculated automatically and exported. Filament volume was divided by total length to differentiate complex and amoeboid-like microglia. Astrocyte morphology was reconstructed manually with automatic volume filling due to the difficulty in automated pathfinding along filaments. Astrocytes were treated as a network for analysis purposes. Total number of filaments, total filament length, and total filament volume were calculated automatically and exported. The degree of space-filling and filament density was determined using the Imaris cell function to isolate discrete areas enclosed by filaments (Membrane Detection algorithm, where the created astrocyte filaments were detected as cell boundaries). Enclosed area sizes and counts were calculated automatically and exported.

### Cytokine Array

Retinas were transferred to HBSS containing protease and phosphatase inhibitors and lysed by ultrasonification (Vibra-Cell). Samples were frozen at −80°C. Samples were thawed and protein was quantified by Bradford assay. Proteome Profiler Mouse XL Cytokine Arrays (R&D Systems) were used according to the manufacturer’s instructions. For each sample, 100 μg of protein was used. Array membranes were imaged on a ChemiDoc iMP system (Bio-Rad) and analyzed by densitometry in FIJI following background subtraction (FIJI; (Schindelin et al., 2012). Spots were analyzed in duplicates and normalized to the reference spots as per the manufacturer’s instructions.

### Statistical Analysis

All statistical analyses were performed in R software. Data were tested for normality with a *Shapiro–Wilk* test. Normally distributed data were analyzed by *Student’s t*-test or *ANOVA* (with *Tukey’s* HSD). Non-normally distributed data were analyzed by a *Kruskal–Wallis* test followed by *Dunn’s* tests with *Benjamini and Hochberg* correction. For microglial morphology, where multiple observations (*i.e.,* individual microglia) come from the same sample (*i.e.,* same retina) a linear mixed effects model was used to reduce intra-class correlation and limit *p* value inflation (Wilson et al., 2017; Tribble et al., 2021a) using the *lme4* package in R (Bates et al., 2015). For protein array data, one-way ANOVA was performed, *p* values were adjusted for multiple comparisons to a false discovery rate (FDR; *q*). Unless otherwise stated, * = *p* < 0.05, ** = *p* < 0.01, ***p < 0.001, NS = non-significant (*p* > 0.05). For box plots, the center hinge represents the median, with upper and lower hinges representing the first and third quartiles; whiskers represent 1.5 times the interquartile range. Violin plots show data distribution by density with accompanying boxplots (as previously mentioned).

## Results

### Retinal Ganglion Cell Axotomy Results in Microglia Neuroinflammation

**FIGURE 1:**
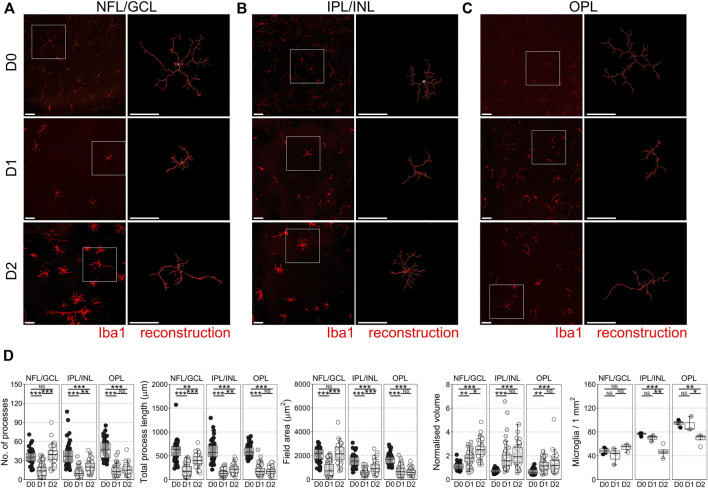
Microglial morphological changes associated with pro-inflammatory phenotypes occur in all retinal microglia niches in retinal explants. Retinal explants were maintained for 1 day (D1) or 2 days (D2) *ex vivo* and compared to D0 controls (eyes fixed immediately after enucleation). IBA1 labeled microglia were imaged and individually reconstructed using Imaris software from the three retinal microglia niches **(A)** nerve fiber layer/ganglion cell layer (NFL/GCL) **(B)** inner plexiform layer/inner nuclear layer (IPL/INL), and **(C)** outer plexiform layer (OPL). **(D)** Across all microglial niches, at D1 there was a significant reduction in the number of microglia processes, total process length, field area, and normalized volume, demonstrating that microglia became less complex and more voluminous, as occurs with a shift towards pro-inflammatory phenotypes. The degree of change was no greater in any one layer, suggesting a global retinal inflammatory environment. There was no significant difference in microglial density within niches demonstrating that there is no active proliferation, migration, or apoptosis of microglia by D1. By D2, microglia in the NFL/GCL had significantly increased number of microglia processes, total process length, field area, and normalized volume, demonstrating an outgrowth of processes reminiscent of microglia in cell culture. This was similar in the IPL/INL, but not in the OPL. Iba1 = microglia specific marker, *n* = 4 retinas for all conditions, scale bars = 50 μm, * = *p* < 0.05, ** = *p* < 0.01, *** = *p* < 0.001, NS = non-significant.

In the mouse retinal axotomy model, enucleation of the eye results in complete axotomy of all retinal ganglion cell (RGC) axons, which drives rapid neurodegeneration from as early as day 1 as the retina is maintained in tissue culture. This includes fragmentation of RGC axons in the nerve fiber layer (NFL), apoptosis of RGCs in the ganglion cell layer (GCL), and atrophy of RGC dendrites in the inner plexiform layer (IPL) (Tribble et al., 2021c). Apoptosis of other retinal neurons does not occur within the first 7 days of explant (Bull et al., 2011; Binley et al., 2016). Microglia reside in three distinct niches in the retina, in the NFL/GCL, IPL, and outer plexiform layer (OPL; photoreceptor synaptic layer) where they perform homeostatic surveillance roles for synaptic and metabolic activity, and for immune or cellular debris antigens. When surveilling, microglia are ramified with numerous low volume processes, but change to a more voluminous, amoeboid morphology when activated. This is associated with a change towards pro-inflammatory and phagocytic phenotypes. By 1 day *ex vivo* (D1) microglia in the NFL/GCL, IPL, and OPL had retracted processes and decreased morphological complexity (Figure 1A–D) suggesting a retina wide inflammatory environment (∼60% average loss of processes, total process length, field area, and ∼60% increase in normalized volume in the NFL/GCL; ∼70% average loss of processes, total process length, field area, and ∼60% increase in normalized volume in the IPL/INL; ∼65% average loss of processes, total process length, field area, and ∼80% increase in normalized volume in the OPL). Microglia counts within niches demonstrated no significant net gain (proliferation or migration), or loss of microglia between layers by D1 (Figure 1D) (*i.e.,* in this context microglia are non-proliferative and non-migratory). By D2, microglia morphology in the NFL/GCL was distinct from that of *in vivo* native or activated phenotypes in retinal neuroinflammation and instead resembled microglia in cell culture (Figures 1A–D). By D2 microglia process number, process length, and field area had increased significantly over D1 and were comparable to D0, suggesting an outgrowth of processes. The processes were thick, as demonstrated by the significant increase in normalized volume over D1 microglia. This also occurred in the IPL/INL but was much less pronounced (Figure 1A–D). In the OPL, there were no detectable significant differences in morphology between D1 and D2 microglia. However, there was a significant decrease in microglia density in the OPL, and IPL/INL (Figure 1D). Together, these suggest a deviation from what would be expected *in vivo* with relevance to the NFL/GCL. We subsequently focused on D1 as a timepoint for the modeling and investigation of early neuroinflammation. We focused on the GCL/NFL as the predominant site of neurodegeneration relevant to RGCs. We tested the anti-neuroinflammatory capacity of VPA in the explant model, and compared this to LPS, a putative positive control for inducing inflammation. At D1, significant RGC death has occurred (33% average loss relative to D0 controls). LPS did not significantly increase RGC death relative to untreated D1 retinas (Supplementary Figure S1). VPA significantly reduced RGC death at D1 compared to D1 untreated retinas, and the remaining density of RGCs was not significantly different from D0 controls (13% average loss relative to D0 controls) (Supplementary Figure S1).

### Valproic Acid Reduces Loss of Microglial Complexity

**FIGURE 2:**
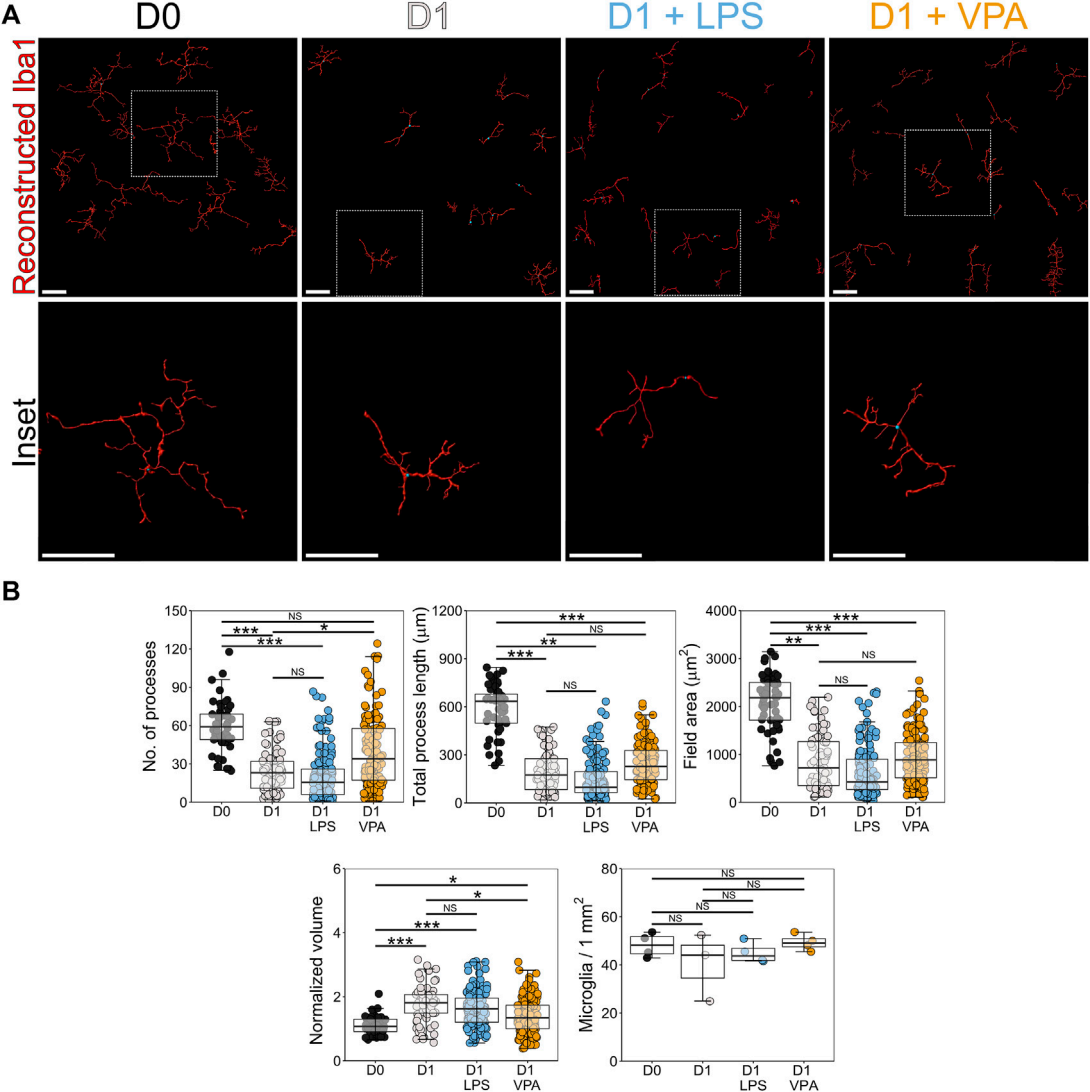
Valproic acid reduces microglial morphological changes associated with pro-inflammatory phenotypes. **(A)** Retinal explants were exposed to LPS, VPA, or untreated and assessed at D1 in comparison to D0 controls. Individual IBA1 labeled microglia were reconstructed using Imaris software. **(B)** At D1 there was a significant reduction in the number of microglia processes, total process length, field area, and normalized volume, demonstrating that microglia became less complex and more voluminous, as occurs with a shift towards pro-inflammatory phenotypes. LPS treatment did not significantly enhance this morphological change. Addition of VPA significantly attenuated these morphological changes, as the number of processes remained higher, and volume was lower than in D1 controls. Total process length and field area were unchanged. There were no significant changes in microglial numbers in retinas exposed to VPA or LPS. These suggest a reduction in the magnitude of a pro-inflammatory shift and a retention of some morphological aspects associated with microglial support functions. *n* = 4 retinas for all conditions, scale bars = 50 μm, * = *p* < 0.05, ** = *p* < 0.01, *** = *p* < 0.001, NS = non-significant.

We next determined whether VPA reduced resident inflammation in the NFL/GCL in response to RGC injury. Individual Iba1 labeled microglia were imaged and reconstructed (Figure 2A). VPA significantly reduced microglial morphological changes but did not completely prevent remodeling (*i.e.,* suggesting a lowered, but not anti-inflammatory, immune cell state). Microglia in VPA treated retinas demonstrated a significantly greater number of processes (40% greater on average) and a significantly lower normalized volume (33% less on average) compared to D1 untreated retinas, without a significant difference in total process length and field area (Figure 2B). This demonstrates that VPA preserved a more branched morphology in microglia (*i.e.,* closer to a surveilling state than an activated state). LPS did not significantly alter microglial morphological changes at D1 relative to untreated D1 retinas (Figure 2B). VPA and LPS did not change microglial density in the NFL/GCL (Figure 2B).

### Valproic Acid Prevents Astrocyte Remodeling

**FIGURE 3:**
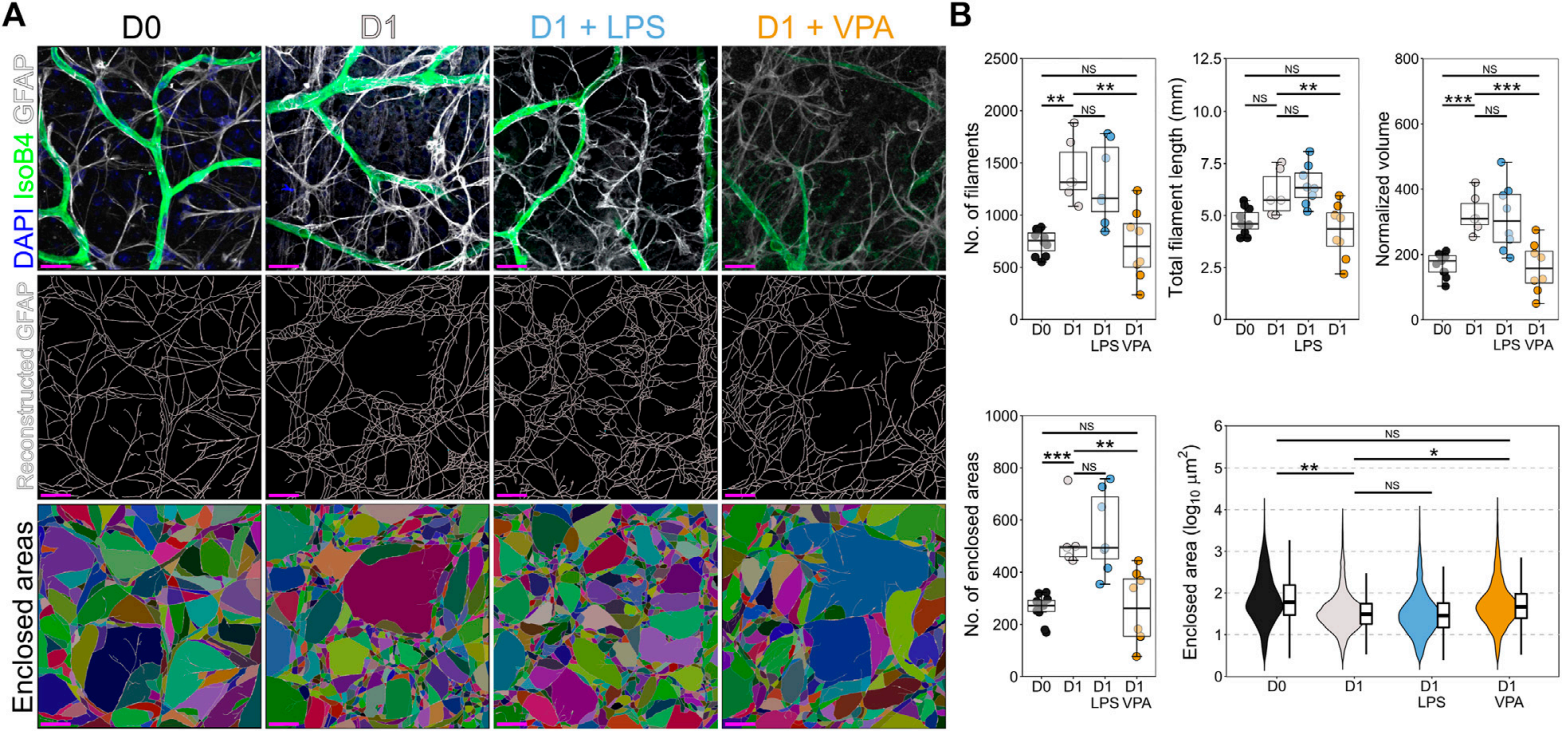
Valproic acid reduces astrocyte morphological changes associated with inflammation and gliosis. **(A)** Retinal explants were exposed to LPS, VPA, or untreated and assessed at D1 in comparison to D0 controls. Individual GFAP fibers were reconstructed to generate astrocyte networks using Imaris software. Areas enclosed by these fibers were generated in Imaris to measure space filling (individual areas are demonstrated by a unique color). **(B)** At D1 there was a significant increase in the number of filaments and the normalized volume, with no significant change in total filament length. This suggests a thickening of GFAP filaments and an increase in filament intersection. Supporting this, the number of enclosed areas was significantly increased, and these were significantly smaller (decreased enclosed area) suggesting an increase in filament volume and space filling by the astrocyte network, as is typical in gliosis. Addition of LPS to the media had no significant effect on astrocyte morphology compared to those in untreated D1 retinas. Addition of VPA significantly attenuated changes to the astrocyte network, with a reduction in the number of filaments, total filament length, and the normalized volume relative to untreated D1 retinas. The number of enclosed areas was significantly reduced, and the area of these was significantly higher than in untreated D1 retinas. There was no significant difference to D0 controls. Together, these suggest a protection against astrocyte morphological change and attenuation of pro-inflammatory and gliotic astrocyte changes. GFAP = astrocyte specific marker in NFL when labeled as flat mounts, *n* = 4 retinas for all conditions, scale bars = 20 μm, * = *p* < 0.05, ** = *p* < 0.01, *** = *p* < 0.001, NS = non-significant. Individual enclosed areas demonstrated by condition as violin plots.

Inflammatory microglial responses are strongly linked to direct activation of astrocytes, and astrocyte upregulation of GFAP (gliosis) is a prominent feature of retinal neurodegeneration. In the retina astrocytes reside only within the NFL where they support RGC axons. Astrocytes also demonstrated remodeling by D1, with increased number and total volume of GFAP fibers (both ∼90% higher on average) in absence of a change to total filament length (Figure 3A,B). This suggests a global thickening of filaments and an increase in small filaments, thus increasing the density of GFAP fibers as is typical of reactive gliosis. Supporting this increase in filament density, astrocyte processes increased space filling of the retina, with a significant decrease in the average area enclosed by crossing filaments (52% less on average), and a significant increase in the number of areas enclosed by crossing filaments (120% higher on average) (Figure 3B). VPA significantly reduced astrocyte remodeling. VPA treated retinas were statistically similar to D0 controls, and VPA also significantly prevented the increase in number of filaments (3% change relative to D0), filament volume (5% change relative to D0), and decreased number and areas enclosed by crossing filaments (0.5% change relative to D0) (Figure 3B). LPS did not significantly alter astrocyte morphological changes at D1 relative to untreated D1 retinas (Figure 3B).

### Valproic Acid Attenuates Pro-inflammatory Cytokine Expression

**FIGURE 4:**
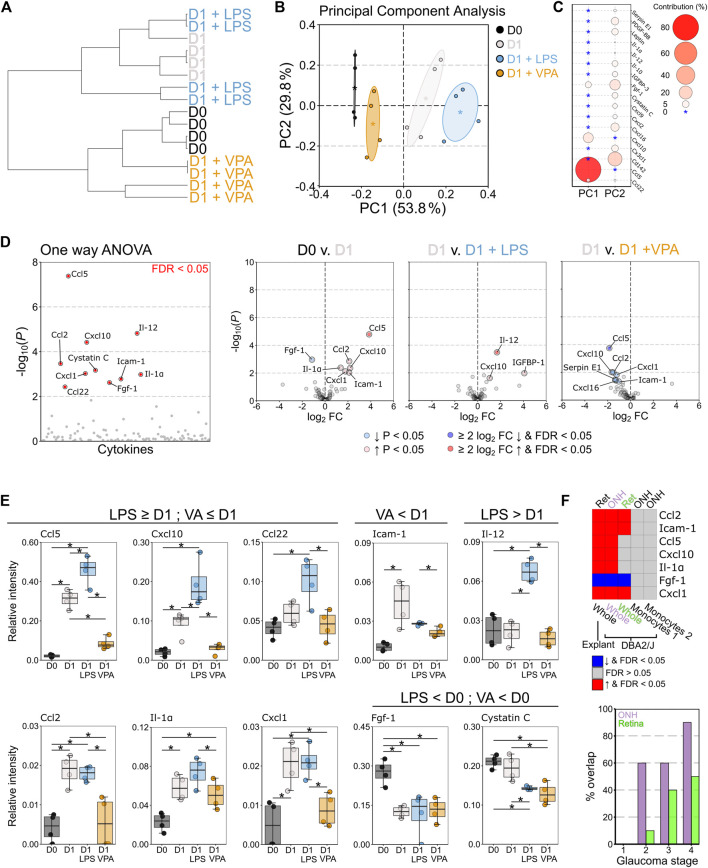
Valproic acid attenuates pro-inflammatory cytokine responses from resident glia. Retinal explants were exposed to LPS, VPA, or untreated and assessed at D1 in comparison to D0 controls. Whole retinal homogenates were probed by cytokine array (111 cytokines and chemokines). **(A)** Hierarchical clustering (*Pearson* correlation) demonstrated that D0 and D1 retinas had discrete global cytokine profiles and that LPS treated retinas were most similar to D1 retinas. VPA treated retinas clustered with D0, demonstrating a protection against global cytokine changes, and a profile most similar to naïve retina. **(B)** Principle component (PC) analysis demonstrated a continuum of change from D0 retina, with D1 VPA least altered, followed by D1, and D1 LPS the most significantly changed. **(C)** This separation was predominantly driven by CCL5 and CXCL10 levels (PC 1) with small contributions from a number of cytokines across PC 2. **(D)** One-way ANOVA demonstrated 10 significantly altered cytokines between conditions (FDR <0.05). Volcano plots for individual comparisons demonstrate significant upregulation of five pro-inflammatory cytokines at D1, with a further increase in three pro-inflammatory cytokines under LPS treatment. VPA treatment significantly reduced six pro-inflammatory cytokines relative to untreated D1 retina **(E)** CCL5, CXCL10, CC122, and IL-1Α demonstrated discrete graded responses by condition where D1 > D0, LPS > D1, and VPA < D1 (and not significantly changed from D0). VPA also reduced ICAM1, but did not affect FGF1 or cystatin **(C)**. LPS significantly enhanced IL12 over all conditions. Collectively, these demonstrate that LPS enhanced the pro-inflammatory response to injury in the retina even if this did not result in detectible morphological change over untreated D1 retinas. VPA demonstrated both a morphological and pro-inflammatory cytokine expression attenuation, but with a greater effect on cytokine profile. **(F)** To validate these findings against an *in vivo* model of RGC injury, significantly changed cytokines between D0 and D1 retina (ret, whole tissue, explant model) were compared to gene array data (ONH/Ret, whole tissue, DBA/2J) and RNA-sequencing data from sorted monocytes (ONH, monocytes, DBA/2J) in the DBA/2J mouse model of glaucoma. Common significant changes and the directionality of change are demonstrated as a heatmap. There was complete overlap with the optic nerve head (ONH) and 4/7 in the retina (ret) when compared to late-stage disease (stage 5 for ONH, four for retina). Monocytes do not differentially express any of these genes (monocytes 1 = enriched for pro-inflammatory changes, monocytes 2 = fewer pro-inflammatory changes), further supporting that the explant model can identify resident glia-specific changes. The % overlap in whole tissue was greatest for later disease stages, and greatest in the ONH, but there was overlap with early stages. This supports that the explant changes reflect real *in vivo* changes from early to late disease. *n* = 4 retinas for all conditions, FC = fold change, FDR = false discovery rate (*q)*, * = *p* < 0.05, ** = *p* < 0.01, *** = *p* < 0.001, NS = non-significant. In B * = group centroid, and the cloud = 95% CI. In **C** * = 0% contribution to a principle component.

VPA treated retina demonstrated some glial morphological changes relative to control retinas which suggests that a mild inflammatory phenotype remained. To better resolve this we profiled a panel of 111 murine chemokines and cytokines to understand the neuroinflammatory changes that occur in response to VPA treatment (Supplementary Dataset S1). Hierarchical clustering demonstrated that VPA treated retinas were most similar to D0 controls, and distinct from D1 untreated, and D1 LPS treated retinas (Figure 4A). Principle component analysis revealed that cytokine profiles were distinct across conditions with a continuum of increasing pro-inflammatory change (Figure 4B) predominantly determined by differences in Ccl5 and Cxcl10 (Figure 4C). One-way ANOVA identified 10 significantly altered cytokines (*q* < 0.05) (Figure 4D). Individual comparisons demonstrated an increase in pro-inflammatory cytokines at D1 compared to D0 control. Addition of LPS significantly increased Ccl5, Cxcl10, and Igfbp-1 relative to D1 untreated retinas and caused a significant increase in Il12, which was not altered in any other condition. Addition of VPA significantly reduced Ccl5, Cxcl10, Ccl2, Cxcl1, and cystatin C relative to D1 untreated controls (Figure 4D). There was no statistically significant difference in Ccl5, Cxcl10, Ccl2, or Icam-1-1 expression between D0 controls and D1 VPA treated retinas, all of which were significantly increased relative to control in D1 untreated retinas (Figure 4E), demonstrating that VPA significantly attenuated pro-inflammatory cytokine expression. However, VPA did not significantly reduce Il1a, nor prevent changes in Fgf1 or cystatin-C relative to D0 controls (Figure 4E). The cytokine changes observed in the explant model (D0 compared to D1 untreated) replicate findings of *in vivo* RGC injury (DBA/2J mouse model of glaucoma (Howell et al., 2011)) in the optic nerve head (ONH) and retina (Figure 4F). None of these genes were differentially expressed in monocytes in the DBA/2J ONH (Williams et al., 2019), further supporting that the changes identified reflected resident glial inflammation (Figure 4F). The % overlap with whole tissue was higher in the ONH than the retina, which occurred earlier in disease in the ONH than the retina, and was highest in later part of the disease in both cases (Figure 4F). This supports the utility of the explant method as a model of RGC injury and the potential to replicate findings *in vivo*.

Taken together, the retinal explant model allows for the assessment of neuroinflammation in a mature tissue system without the caveat of a systemic immune response (*i.e.,* monocyte infiltration) with which to explore resident glia responses. Using this model, we identify VPA as a potent retinal anti-neuroinflammatory agent that provides RGC neuroprotection even in context of severe acute insult.

## Discussion

The retinal explant model has previously been demonstrated to be a useful tool for the rapid exploration of neurodegenerative and neuroprotective mechanisms in mouse, rat, pig, and human retina (Bull et al., 2011; Bell et al., 2016; Binley et al., 2016; Osborne et al., 2016; Williams et al., 2017a; Tribble et al., 2021c; Enz, Tribble and Williams, 2021). Our data demonstrate that it is also highly amenable in investigating neuroinflammation without the loss of spatial interaction that occurs in cell culture or direct insult to glial and astrocytes that may occur from cutting brain slices for culture (as, in this model, only retinal ganglion cell axons are axotomized). However, the utility of this model may be limited to short-term investigation of RGC relevant insult since changes at D2 resembled cell culture-like outgrowth more than *in vivo* inflammatory changes. Inflammation could be successfully modulated within a 1 day time window by the addition of test compounds to the media. Using this model, we demonstrated that VPA provides significant protection against neuroinflammation. VPA significantly attenuated pro-inflammatory cytokine release and reduced glial remodeling caused by retinal ganglion cell axotomy. As a putative positive control, retinas were treated with LPS. LPS further exacerbated inflammation at D1, although this was not a strong effect. There was high overlap with inflammatory changes identified in the explant and the DBA2/J mouse model of glaucoma (Howell et al., 2011), at early and late molecular stages of disease, suggesting that this model recapitulates *in vivo* retinal inflammation well. Greater overlap in the ONH than retina reflects that the primary insult is to retinal ganglion cell axons (as the ONH is enriched for retinal ganglion cell axons). That overlap was greater in the later molecular disease stages and reflects the acute insult of the explant model. Significantly, none of the genes identified are differentially expressed by monocytes in early disease stages, further supporting that the explant model can identify resident glia-specific changes.

VPA has previously demonstrated neuroprotection of RGCs in optic nerve crush, normal tension glaucoma, and optic neuritis (Biermann et al., 2010; Kimura et al., 2015; Liu et al., 2017). VPA treatment of optic neuritis, reduced optic nerve mRNA expression of TNF-α and Il-1β, inhibited upregulation of phosphorylated NF-κB p65 and downregulation of IκB suggesting a suppression of the NF-κB signaling pathway (Liu et al., 2017). The NF-κB signaling pathway is a major driver of pro-inflammatory cytokine expression. VPA has been demonstrated to inactivate the NF-κB pathway inflammatory response by inhibiting histone deacetylase (HDAC) 3, which increases STAT1 and NF-κB acetylation (inactivating NF-κB signalling; (Chen S et al., 2018)). VPA is a broad HDAC inhibitor, with reported inhibition of HDAC1 and 2 (Class Ia), HDAC 3 (Class Ib), HDAC 8 (Class Ic), and HDAC 4, 5, and 7 (Class IIa) resulting in epigenetic inhibition of cell cycle, cell differentiation, and apoptotic genes (although the effects are cell type specific (Soria-Castro et al., 2019)). Administration of VPA to microglia *in vitro* results in apoptosis (Chen et al., 2007), although this has not been reported in animal models where neuroinflammation is a feature (*i.e.,* this may purely be a feature of isolated microglia outside of a mature tissue context). We observed no significant change in microglia numbers in VPA treated retinas which further support the need to explore inflammation in the context of *in vivo* or *ex vivo* systems where cell interactions remain intact. VPA improved RGC survival which could be a result of both HDAC mediated suppression of RGC apoptosis and a reduction in neuroinflammation. Broad and targeted inhibition of HDACs has demonstrated significant protection against apoptosis in RGCs (Biermann et al., 2010; Pelzel, Schlamp and Nickells, 2010; Chindasub et al., 2013), although targeted deletion of specific HDCAs (1-3) does not protect against injury-induced gene silencing (Schmitt et al., 2014), suggesting that the neuroprotective effects of broad-spectrum inhibitors such as VPA act through multiple mechanisms. Whether this includes suppression of glial inflammation is yet to be determined *in vivo*. Future comparison of the anti-inflammatory properties of VPA to other HDAC inhibitors (*e.g.* trichostatin A, sodium butyrate) would allow for the development of targeted HDCA inhibition of glial neuroinflammatory responses.

VPA significantly attenuated RGC apoptosis and inflammation in the explant model. Since we observed no clear difference in the degree of microglial activation between the retinal layers, this suggests that the degree of neuron insult/death is not the determinant of the degree of inflammation (since only RGCs are injured and only RGCs have degenerated by D1). This suggests that inflammation was suppressed as a direct glial effect, rather than as secondary to improved RGC survival. We cannot preclude survival bias which would require definitive testing (*e.g.* where RGCs do not degenerate such as in mice carrying *Bax* ^*−/−*^ alleles and/or the Wallerian degeneration slow allele (*Wld* ^*S*^) in RGCs, or targeted HDAC inhibition of glia). VPA inhibition of HDAC 3 also upregulates the Nrf2/ARE pathway, promoting autophagy and antioxidative responses (Chen X et al., 2018). Owing to its simple chemical structure, VPA may also modulate metabolism, particularly the use of glucose, fatty acids, and glutamine in the brain. Chronic administration of VPA drastically reduces brain glucose consumption (Leiderman et al., 1991). Shifting retinal metabolism away from glucose using a ketogenic diet is both neuroprotective and anti-inflammatory in glaucoma (Harun-Or-Rashid et al., 2018; Harun-Or-Rashid and Inman, 2018). These support multiple mechanisms of neuroprotection and anti-inflammation action of VPA.

## Conclusion

The retinal explant model is a useful tool to explore resident neuroinflammation. Neuroinflammation can be modulated and explored on a rapid timescale whilst maintaining a complex system of cell interactions. VPA can attenuate resident glial inflammation in the retina and is a useful tool to further explore inhibition of pro-inflammatory glia in neurodegenerative disease.

## Data Availability

The original contributions presented in the study are included in the article/Supplementary Materials, further inquiries can be directed to the corresponding author.

## Supplementary Material

Supplementary Figure S1 Valproic acid provides robust protection against retinal ganglion cell death following axotomy. **(A)** Using a retinal explant model, where retinas are maintained in tissue culture following eye enucleation, the effect of Valproic acid (VPA) and Lipopolysaccharide (LPS) on retinal ganglion cell (RGC) survival were assessed. **(B)** At 1 day *ex vivo* (D1), significant retinal ganglion cell death has occurred compared to naïve control retina (D0, eyes fixed immediately following enucleation). Addition of LPS to the culture media did not significantly increase RGC death at D1 (D1 + LPS). Addition of VPA significantly reduced RGC death at D1 (D1 + VPA). RBPMS = RGC specific marker, *n* = 6 retinas for all conditions, scale bars = 20 µm, * = *P* < 0.05, ** = *P* < 0.01, *** = *P* < 0.001, NS = non-significant.
