# Supplementary figures and images for: Valproic Acid Reduces Neuroinflammation to Provide Retinal Ganglion Cell Neuroprotection in the Retina Axotomy Model
Source: Front Cell Dev Biol. 2022 May 12;10:903436. doi: 10.3389/fcell.2022.903436 (PMC9135180; doi:10.3389/fcell.2022.903436)

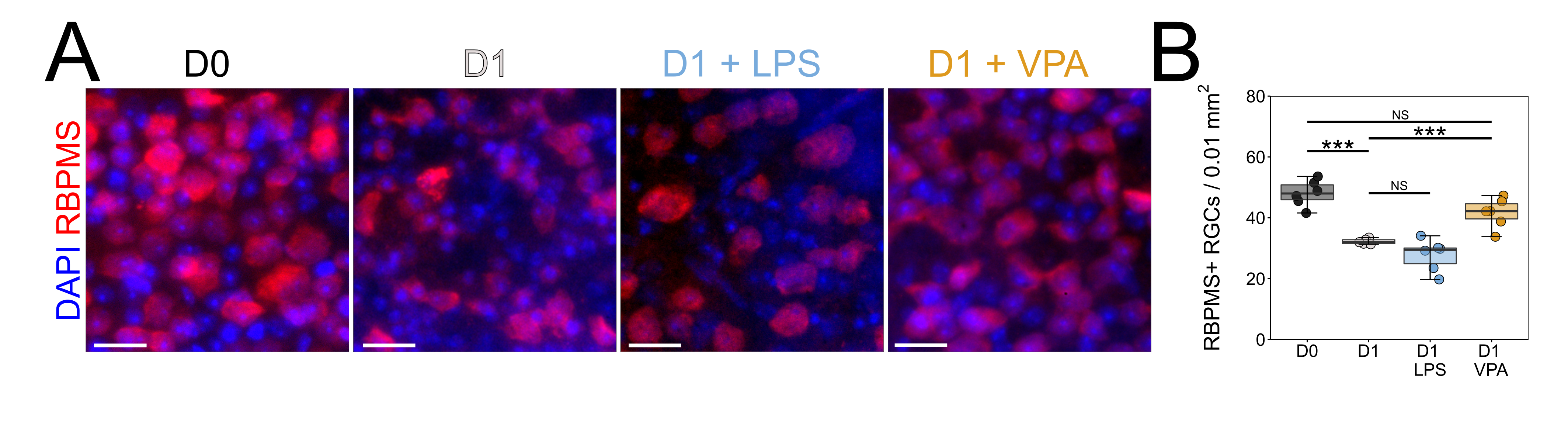

Supplement: Supplementary file 1 [file Image1.TIF]
